# The Study of Electrical Properties for Multilayer La_2_O_3_/Al_2_O_3_ Dielectric Stacks and LaAlO_3_ Dielectric Film Deposited by ALD

**DOI:** 10.1186/s11671-017-2004-1

**Published:** 2017-03-29

**Authors:** Xing-Yao Feng, Hong-Xia Liu, Xing Wang, Lu Zhao, Chen-Xi Fei, He-Lei Liu

**Affiliations:** Key Laboratory for Wide-Band Gap Semiconductor Materials and Devices of Education, School of Microelectronics, Xidian University, Xi’an, 710071 China

## Abstract

The capacitance and leakage current properties of multilayer La_2_O_3_/Al_2_O_3_ dielectric stacks and LaAlO_3_ dielectric film are investigated in this paper. A clear promotion of capacitance properties is observed for multilayer La_2_O_3_/Al_2_O_3_ stacks after post-deposition annealing (PDA) at 800 °C compared with PDA at 600 °C, which indicated the recombination of defects and dangling bonds performs better at the high-*k*/Si substrate interface for a higher annealing temperature. For LaAlO_3_ dielectric film, compared with multilayer La_2_O_3_/Al_2_O_3_ dielectric stacks, a clear promotion of trapped charges density (*N*_ot_) and a degradation of interface trap density (*D*_it_) can be obtained simultaneously. In addition, a significant improvement about leakage current property is observed for LaAlO_3_ dielectric film compared with multilayer La_2_O_3_/Al_2_O_3_ stacks at the same annealing condition. We also noticed that a better breakdown behavior for multilayer La_2_O_3_/Al_2_O_3_ stack is achieved after annealing at a higher temperature for its less defects.

## Background

With the continuous development of integrated circuit, high-*k* materials have been extensively studied to substitute traditional SiO_2_ gate dielectrics in CMOS devices as a solution for the saturation of the leakage current and power consumption [1–3]. Lanthanum oxide (La_2_O_3_), aluminum oxide (Al_2_O_3_), yttrium oxide (Y_2_O_3_), hafnium oxide (HfO_2_), and zirconium oxides (ZrO_2_) have been tried to use as alternative gate dielectric materials [4–7]. Among them, La_2_O_3_ is regarded as a promising candidate due to the high dielectric constant (*k* ~ 27) and large band gap. Simultaneously, the accompanying problems also draw great attentions [8, 9].

The electrical properties of La_2_O_3_ and Al_2_O_3_ dielectric stacks have been studied by many researchers. Srikant Jayanti pointed out that significant improvement about charge trapping and leakage characteristics was obtained by using a La_2_O_3_ interface scavenging layer for Al_2_O_3_ interpoly dielectric [10]. Lee found that the hydration of La_2_O_3_ can be blocked by the Al_2_O_3_ in Al_2_O_3_/La_2_O_3_/Si (ALO structure) after the annealing treatment at 700 °C [11]. Researchers also revealed that the ultra-thin 0.5-nm Al_2_O_3_ inserted layer under the 4 nm LaAlO_3_ can reduce the EOT to 1.2 nm with optimized interface trap density. And compared with La_2_O_3_ and Al_2_O_3_ dielectric stacks (ALO or LAO structure), the lanthanum aluminate (LaAlO_3_) meets the thermal processing requirement better, since the added Al_2_O_3_ greatly improves the chemical stability and crystallization temperature [12, 13]. However, the electrical property difference between the La_2_O_3_/Al_2_O_3_ dielectric stacks and LaAlO_3_ have not been fully studied. In this paper, multilayer La_2_O_3_/Al_2_O_3_ stacks and LaAlO_3_ dielectric film were prepared by ALD reactor, and then, post-deposition annealing (PDA) was carried out at different temperatures. After the deposition of metal gate, the interfacial issues and electrical properties of the fabricated MIS structures were studied.

## Methods

P-type Si (100) wafers with resistivity of 3–8 Ω cm were dipped in deionized water and diluted HF for 3 min, respectively, to remove the native oxide before deposition. Then La_2_O_3_/Al_2_O_3_ high-*k* stacks were deposited on Si wafers by ALD reactor (Picosun R-150, Espoo, Finland) in 300 °C. La(i-PrCp)_3_ and trinethyluminium (TMA) were used as precursors of La and Al, and O_3_ was used as oxidant. Besides, ultra-high purity nitrogen (N_2_, 99.999%) was employed as purge gas and carrier. The rapid thermal annealing (RTA) process was carried out at 600 and 800 °C in N_2_ ambient for 1 min after the deposition. A metal electrode with a diameter of 300 μm was fabricated by depositing 150 nm Al by the electron-beam evaporation through a shadow mask. In the end, the electrical properties including capacitance-voltage (*C*-*V*), conductance-voltage (*G*-*V*), and leakage current-voltage (*I*-*V*) characteristics were evaluated using an Agilent B1500A semiconductor parameter analyzer at the frequency of 100 kHz. X-ray photoelectron spectroscopy (XPS) was used to examine the bonding structures and chemical quantitative composition of the films. C1s peak from adventitious carbon at 284.6 eV [14] was used as an internal energy reference during the analysis.

## Results and Discussion

**Fig. 1:**
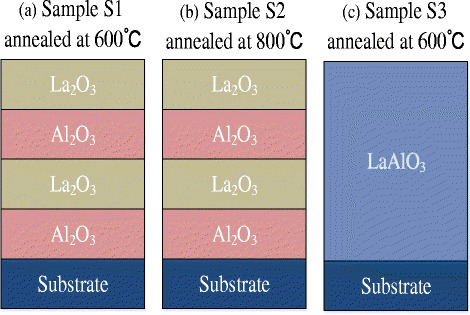
Schematic structures of multilayer La_2_O_3_/Al_2_O_3_ stack samples S1 and S2 and LaAlO_3_ sample S3

The schematic structures and annealing temperatures are shown in Fig. 1 and Table 1. In Table 1, one-cycle La_2_O_3_ or Al_2_O_3_ came out from the reaction of a pulse of La or Al precursor and a pulse of oxidant O_3_. The samples S1 and S2 are multilayer La_2_O_3_/Al_2_O_3_ stacks with the same film structure and with 600 and 800 °C annealing temperatures, respectively, while the sample S3 is the LaAlO_3_ dielectric film annealed at 600 °C.

**Table 1:** The structures and annealing temperatures of samples S1–S3

Sample	Film structures	Annealing temperature
S1	2 × (20-cycle Al_2_O_3_ + 20-cycle La_2_O_3_)	600 °C
S2	2 × (20-cycle Al_2_O_3_ + 20-cycle La_2_O_3_)	800 °C
S3	40 × (1-cycle Al_2_O_3_ + 1-cycle La_2_O_3_)	600 °C

**Fig. 2:**
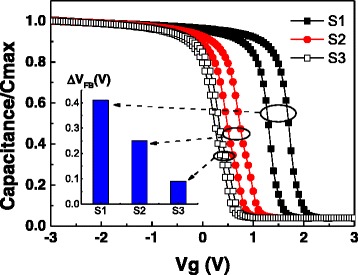
*C*-*V* curves of samples S1–S3. Δ*V*_FB_ were extracted from *C*-*V* curves

**Fig. 3:**
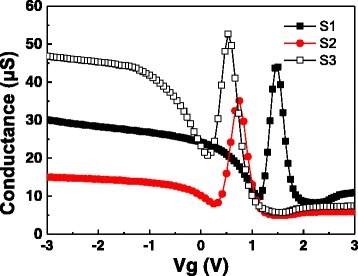
*G*-*V* curves of samples S1–S3

Figures 2 and 3 show the *C*-*V* and *G*-*V* curves of samples S1, S2, and S3. The capacitors were swept forward (bias from negative to positive) and backward (bias from positive to negative) to check the *C*-*V* hysteresis at the frequency of 100 kHz. *G*-*V* curves were obtained simultaneously with the *C*-*V* curves. The Δ*V*_FB_ is the flat band voltage difference of the *C*-*V* curve and its hysteresis. A clear decreasing of Δ*V*_FB_ was observed with a higher annealing temperature with the same multilayer La_2_O_3_/Al_2_O_3_ stack structure. More apparently, sample S3 has a very small Δ*V*_FB_ compared with S1 and S2.

As we know, the trapped charges are responsible for the Δ*V*_FB_ (hysteresis width) [15], and we assume that the two-dimensional distribution of traps near the interface contributes to the film capacitance. Then, the trapped charges density (*N*_ot_) can be expressed as in the following equation [16, 17]:

1 $$ {N}_{\mathrm{ot}}=\frac{\varDelta {V}_{\mathrm{FB}}{C}_{\mathrm{ox}}}{qA} $$

2 $$ {C}_{\mathrm{ox}}={C}_{\mathrm{ac}}\left[1+{\left(\frac{G_{\mathrm{ac}}}{\omega {C}_{\mathrm{ac}}}\right)}^2\right] $$

Where *C*_ox_ is the insulator capacitance, *q* is the electron charge (1.602 × 10^−19^ C), *A* is the electrode area, *C*_ac_ is the measured accumulation capacitance, *ω* is the angular frequency, and *G*_ac_ is the conductance in accumulation region. By this model, the *N*_ot_ is estimated to be 2.46 × 10^12^ cm^−2^, 1.54 × 10^12^ cm^−2^, and 6.20 × 10^11^ cm^−2^ for samples S1, S2, and S3 respectively.

The interface trap density (*D*_it_) value is another characteristic to evaluate the interface property of fabricated MIS capacitors. By Hill-Coleman single-frequency approximation, the *D*_it_ can be expressed as [18]:

3 $$ {D}_{\mathrm{it}}=\frac{2}{qA}\frac{\frac{G_{\mathrm{ac}}}{\omega}}{\left[{\left(\frac{G_{\max }}{\omega {C}_{\mathrm{ox}}}\right)}^2+{\left(1-\frac{C_{\mathrm{c}}}{C_{\mathrm{ox}}}\right)}^2\right]} $$

Where *G*_max_ is the maximum value of conductance, and *C*_c_ is the corresponding capacitance of the gate voltage at which the *G*_max_ is obtained. The *D*_it_ of samples S1, S2, and S3 can be figured out as 1.24 × 10^12^ eV^−1^cm^−2^, 6.05 × 10^11^ eV^−1^cm^−2^, and 1.98 × 10^12^ eV^−1^cm^−2^ respectively. A higher *D*_it_ of sample S1 than S2 can be attributed to the more recombination of dangling bonds at the high-*k*/Si interface for a higher annealing temperature. Compared with S1, sample S3 contains more La_2_O_3_/Al_2_O_3_ interfaces (we can regard the LaAlO_3_ dielectric film as a multilayer La_2_O_3_/Al_2_O_3_ stack which contains a very large number of plies), which means more interface trap.

So, a significant promotion in these two electrical properties can be obtained for a multilayer La_2_O_3_/Al_2_O_3_ stack at 800 °C annealing temperature compared with 600 °C. However, for LaAlO_3_ dielectric film, a promotion of *N*_ot_ and a degradation of *D*_it_ are obtained simultaneously. In a more comprehensive perspective, a better capacitance property are obtained from the LaAlO_3_ dielectric film, since the lower flat band voltage and less Δ*V*_FB_. And it is worth noting that a flat band voltage modulation can be carried out by manipulating the annealing temperature and the number of plies in multilayer La_2_O_3_/Al_2_O_3_ stack [19].

**Fig. 4:**
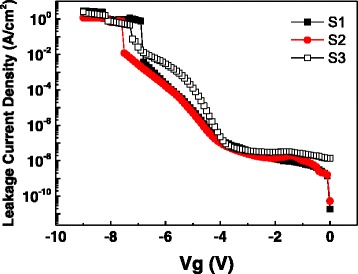
*I*-*V* curves of samples S1–S3

**Fig. 5:**
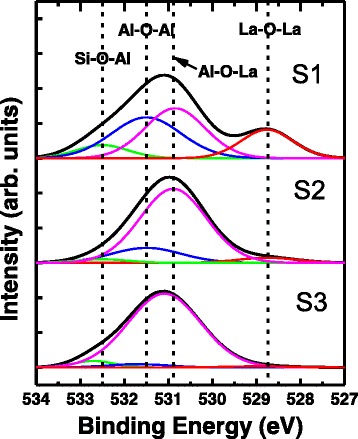
O1s XPS spectra of samples S1–S3. The O1s spectra were fitted with four peaks (Si–O–Al, Al–O–Al, Al–O–La, and La–O–La)

Figure 4 shows the leakage current density as a function of the applied gate voltage. S1 and S2 show a very similar leakage current, while S3 shows a 1 ~ 2 orders of magnitude larger leakage current with the same applied gate voltage. Then, XPS was employed to seek the explanation. Figure 5 shows the O1s XPS spectra of samples S1–S3, which was fitted with four peaks Si–O–Al (532.5 eV), Al–O–Al (531.5 eV), Al–O–La (530.9 eV), and La–O–La (528.75 eV). It is obvious that La–O–Al peaks become larger, while La–O–La, Al–O–Al, and Si–O–Al peaks become smaller from S1 to S3. Therefore, compared with S1 and S2, more La_2_O_3_ will appear at the interface of high-*k*/Si in sample S3. La_2_O_3_ has lower conduction band offset (CBO) and valence band offset (VBO) with respect to p-type Si substrate compared with Al_2_O_3_ (the CBO and VBO are about 2.3 and 2.6 eV for La_2_O_3_ and are about 2.8 and 4.9 eV for Al_2_O_3_) [20]. So, the increase of La_2_O_3_ in the high-*k*/Si interface will lead to the decrease of band offset as well as the increase of leakage current.

In addition, we notice that the sample S2 has a higher breakdown voltage than S1. It can be attributed to the lower trapped charges density, since structural defects lead to the possibility to generate a conduction path in gate dielectric [15].

## Conclusions

In summary, the capacitance and leakage current properties for multilayer La_2_O_3_/Al_2_O_3_ stacks and LaAlO_3_ dielectric film have been studied systematically. A clear promotion of capacitance properties is observed for multilayer La_2_O_3_/Al_2_O_3_ stacks after PDA at 800 °C compared with that at 600 °C. As for LaAlO_3_ dielectric film, compared with multilayer La_2_O_3_/Al_2_O_3_ dielectric stacks, a promotion of *N*_ot_ and a degradation of *D*_it_ can be obtained at the same time. On the other hand, the LaAlO_3_ dielectric film presents a better leakage property which attributes to its higher CBO and VBO with respect to p-type Si substrate. And the breakdown behavior showed a clear improvement for the film with a higher annealing temperature for its less defects.

## Abbreviations

ALD: Atomic layer deposition
CBO: Conduction band offset
CMOS: Complementary metal oxide semiconductor
*C*-*V*: Capacitance-voltage
*D*_*it*_: Interface trap density
*G*-*V*: Conductance-voltage
*I*-*V*: Leakage current-voltage
*N*_ot_: Trapped charges density
PAD: Post-deposition annealing
RTA: Rapid thermal annealing
TMA: Trinethyluminium
VBO: Valence band offset
XPS: X-ray photoelectron spectroscopy
